# Brain Biochemistry and Personality: A Magnetic Resonance Spectroscopy Study

**DOI:** 10.1371/journal.pone.0026758

**Published:** 2011-11-03

**Authors:** Sephira G. Ryman, Chuck Gasparovic, Edward J. Bedrick, Ranee A. Flores, Alison N. Marshall, Rex E. Jung

**Affiliations:** 1 The Mind Research Network (MRN), University of New Mexico Health Sciences Center, Albuquerque, New Mexico, United States of America; 2 Department of Internal Medicine, University of New Mexico Health Sciences Center, Albuquerque, New Mexico, United States of America; 3 Department of Neurosurgery, University of New Mexico Health Sciences Center, Albuquerque, New Mexico, United States of America; The University of Hong Kong, Hong Kong

## Abstract

To investigate the biochemical correlates of normal personality we utilized proton magnetic resonance spectroscopy (^1^H-MRS). Our sample consisted of 60 subjects ranging in age from 18 to 32 (27 females). Personality was assessed with the NEO Five-Factor Inventory (NEO-FFI). We measured brain biochemistry within the precuneus, the cingulate cortex, and underlying white matter. We hypothesized that brain biochemistry within these regions would predict individual differences across major domains of personality functioning. Biochemical models were fit for all personality domains including Neuroticism, Extraversion, Openness, Agreeableness, and Conscientiousness. Our findings involved differing concentrations of Choline (Cho), Creatine (Cre), and N-acetylaspartate (NAA) in regions both within (i.e., posterior cingulate cortex) and white matter underlying (i.e., precuneus) the Default Mode Network (DMN). These results add to an emerging literature regarding personality neuroscience, and implicate biochemical integrity within the default mode network as constraining major personality domains within normal human subjects.

## Introduction

There is increasing interest within the neurosciences regarding the relationship between normal personality traits and brain structure. The Big Five personality model quantifies relatively distinct personality traits including the tendencies: 1) to experience negative emotions–Neuroticism; 2) the tendency to be assertive, to be sociable, and to experience positive emotions–Extraversion; 3) to be imaginative and unconventional–Openness; 4) to be trustworthy and cooperative–Agreeableness; and 5) to be organized and reliable–Conscientiousness [1]. Importantly, normal personality traits have been well linked to clinical disorders such as schizoid, avoidant, and borderline personalities [2] as well as clinical psychiatric disorders, particularly major depression [3], [4] and schizophrenia [5].

Several studies have linked normal personality traits to discrete brain regions in normal human cohorts [6], [7], [8], [9], [10]. Both increased and decreased brain volumes were associated with personality variables, particularly Extraversion and Neuroticism, suggesting a complex interplay of excitatory and inhibitory networks underlying normal personality functioning. To investigate the biochemical correlates of the main personality factors we utilize proton magnetic resonance spectroscopy (^1^H-MRS), an imaging technique that allows for the assay of neurochemistry *in vivo.* At low field strengths, and relatively long echo times, metabolites that are reliably measured with ^1^H-MRS include N-acetylaspartate (NAA), creatine (Cre), and choline-containing compounds (Cho). Our group and others have previously demonstrated relationships between spectroscopic measures of metabolite concentration and measures of intelligence [11], [12], [13], [14], [15], [16], [17], affect [18], and creativity [19], in cohorts of normal human subjects.

Previous studies have demonstrated a wide range of cortical and subcortical regions implicated in personality functioning, although regions within the medial prefrontal cortex [7], [10], [20] and posterior cingulate cortex [8] have been repeatedly implicated across studies. We hypothesize that personality variables from the Big Five will relate to spectroscopic measures within the cingulate gyrus and white matter regions projecting to the frontal and parietal lobes.

## Methods

### Sample

Written informed consent was obtained from all subjects prior to participation in the experimental protocol. The consent form was approved by the institutional review board of the University of New Mexico, and consistent with the Declaration of Helinski. The sample consisted of 66 subjects, recruited from postings at the University of New Mexico, who ranged in age from 18 to 32 (33 males, 27 females). Participants were screened by a clinical neuropsychologist (R.E.J.) and determined to be free of neurological and psychological disorders that would impact experimental hypotheses (e.g., major depression, attention deficit disorder, prior traumatic brain injury). Participants were also screened for conditions that would prohibit undergoing a Magnetic Resonance (MR) scan (e.g., metal implant, orthodontic braces, and severe claustrophobia). Six subjects were removed from the experimental sample after they were determined to have spectroscopic data which was below quality thresholds (as determined by Cramer Rao lower bounds >20% on NAA), leaving a final experimental sample of 60. This sample has previously been reported [11], [19].

### Behavioral Measures

Personality was assessed with the NEO Five-Factor Inventory (NEO-FFI). The NEO-FFI is a self-administered measure of normal personality functioning, which produces summary scores across five domains: Neuroticism, Extraversion, Openness, Agreeableness, and Conscientiousness. The NEO-FFI scales show correlations of .75 to .89 with the NEO-PI validimax factors. Internal consistency values range from .74 to .89 [1].

### Spectroscopic acquisition

MR examinations were performed on a 1.5T Siemens Sonata scanner using an eight-channel phased array head coil. MR spectroscopy was obtained using two-dimensional (2D) Chemical Shift Imaging (CSI) prescribed from the supraventricular slice of a T1-weighted sagittal image [field of vision  = 200×200 mm, repetition time/echo time  = 1500/135 ms, voxel size  = 8.3×8.3×15.0 mm, acquisition time 9∶32 (min:sec). The PRESS CSI volume of interest (VOI) was prescribed immediately above the lateral ventricles and parallel to the anterior commissure-posterior commissure line. The size of the VOI was ∼75±5 mm left to right and 90±5 anterior to posterior. Both water suppressed and water unsuppressed spectra were acquired.

### Analysis

**Figure 1:**
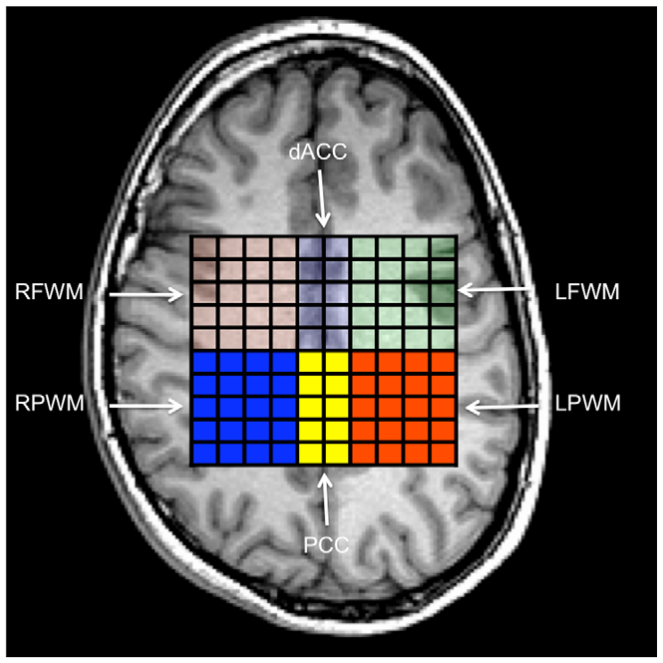
Axial view of MRSI grid comprising the six regions of interest (averages of individual 0.6 cm^3^ voxels within each region). Image is in radiological convention (left side of image, right hemisphere). Going left to right from top to bottom: right frontal white matter (RFWM), dorsal dACC cortex (dACC), left frontal white matter (LFWM), right parietal white matter (RPWM), PCC cortex (PCC), and left parietal white matter (LPWM).

Spectra were zero-filled to 32×32 points in k-space, applying a Hamming filter with a 50% window width, and 2D spatial Fourier transformation (FT). LCmodel was used for post processing of spectra [21]. Tissue segmentation allowed calculation of the exact proportion of grey and white matter in the supraventricular area of interest and correction of metabolic values based on percentage of tissue values (grey, white, and cerebrospinal fluid)[22]. Six regions within the supraventricular area (Figure 1) were determined by an automated script created in house. This script first determined the dimensions of the spectroscopic voxel of interest (e.g., 10×8), and then divided the VOI into six regions corresponding to left frontal white matter (LFWM), right frontal white matter (RFWM), dorsal anterior cingulate cortex (dACC, Brodmann areas 24/32), left parietal white matter (LPWM), right parietal white matter (RPWM), and posterior cingulate cortex (PCC, Brodmann area 31).

### Statistical analysis

Personality scores (Neuroticism, Extraversion, Openness, Agreeableness, Conscientiousness) were regressed on three metabolites concentrations (NAA, Cho, Cre) from the six voxel locations. We conducted stepwise and backward multiple regressions, controlling for age, sex, and handedness before engaging in model selection via the bias-corrected Akaike Information Criterion (AICc) [23]. Stepwise and backward linear regression analyses were conducted in SPSS (Version 16.0 for Windows). AICc was computed for all possible models in R Version 2.13.1. The significance of interactions was assessed after the best models with main effects were identified. Composite spectroscopic measures of NAA, Cre and Cho in each of the six major regions served as independent variables and each of the Big Five Dimensions (composite scores from the NEO-FFI) separately served as the dependent measures. Due to the high correlation of metabolites both within and across brain regions, no correction for multiple comparisons was made to avoid Type II error. Scatter plots, with two-tailed Pearson r correlations, were used to display the relationships between each of the statistically significant metabolite and the corresponding personality dimension.

## Results

Participants were young adults (23.45 +/− 3.748), well matched by sex (33 males, 27 females) and handedness (males 31 right, 2 left; females 27 right, 3 left). The mean FSIQ was above average for both sexes (males 118; females 117). Table 1 shows the demographic statistics and personality measures.

**Table 1:** Demographic variables for the sample.

*Measure*	*Mean*	*SD*
Age	23.45	3.75
FSIQ	117.8	9.75
Neuroticism	16.43	7.37
Extraversion	30.65	6.89
Openness	34.93	5.47
Agreeableness	31.15	5.14
Conscientiousness	33.13	6.39

Table legend: SD  =  standard deviation; FSIQ  =  Full Scale Intelligence Quotient.

Stepwise regression found that parietal white NAA predicted Neuroticism (R**^2^** = .31, β = .27); backward regression found that a model including lower PCC Cho (β = −.31) and higher LPWM Cho (β = .34) predicted Neuroticism (R**^2^** = .36). The interaction of these variables was not significant (β = −.24). Model selection by AICc (Table 2) found a combination of NAA and Cho variables, obtained from stepwise and backwards regressions to best fit Neuroticism.

**Table 2:** Model Selection by Various Statistical Methods.

*Method*	*Neuroticism*	*Extraversion*	*Openness*	*Agreeableness*	*Conscientiousness*
Stepwise	NAA4 β = .27	CRE4 β = −.31		CRE4 β = −.43	CHO3 β = −.38
α = 0.05				NAA5 β = −.27	CRE5 β = .35
	r^2^ = .31	r^2^ = .12		r^2^ = .35	r^2^ = .25
Interaction				ns, β = −1.58	ns, β = −.57
Backward	CHO5 β = −.31	CRE2 β = .49		CRE4 β = −.43	CHO3 β = −.38
α = 0.05	CHO6 β = .34	CHO2 β = −.61		NAA5 β = −.27	CRE5 β = .35
		CHO5 β = .36			
	r^2^ = .36	r^2^ = .17		r^2^ = .35	r^2^ = .25
Interaction	ns, β = −.24	ns, β = .02/.19/−.56		ns, β = −1.58	ns, β = −.57
AICc 1	NAA4	NAA2	NAA1	NAA5	CHO3
	CHO5	CRE2	NAA4	CRE2	CRE5
	CHO6	CHO2	CHO3	CRE4	(205.08)
	(210.21)	CHO5	(197.37)	(173.80)	
		(220.96)			
AICc 2	CHO5	NAA2	NAA1	CRE4	CHO3
	CHO6	CHO2	NAA4	CRE5	CRE5
	(201.51)	CHO5	CHO2	(174.01)	CHO2
		(221.47)	(197.51)		(206.50)
AICc 3	NAA4	NAA2	CHO2	NAA5	NAA2
	CHO4	NAA5	(197.66)	CRE2	CHO3
	CHO5	CRE4		CRE4	CRE5
	CHO6	CHO2		CHO2	(206.94)
	(212.01)	CHO5		CHO3	
		(222.07)		(174.08)	

Models selected by various statistical methods. Columns are individual response variables. All models include age, gender and handedness as covariates. Cell entries are variables selected from 18 metabolite-region combinations. If a cell is empty then no other variables are added. AICC 1, 2 and 3 are the three best models selected by the adjusted Akaike Information Criterion (AICC), which is widely viewed as the best model selection criteria available. This measure is penalized version of minus twice the maximized log-likelihood, where the penalty is a function of the number of predictors in the model. The value of AICC is given in parentheses.

A model including dACC Cre (β = .49) and Cho (β = −.61), as well as PCC Cho (β = .36) predicted Extraversion using backward regression (R**^2^** = .17). No pairwise interaction of predictor variables were significant. AICc found this model for Extraversion to be underfitted, also including dACC NAA in all the top models.

No variables were selected for predicting Openness in stepwise or backward regression. The best models based on AICc used RFWM NAA (β = −.47), RPWM NAA (β = .42) and dACC Cho to predict Openness to experience ( R**^2^** = .16).

Both stepwise and backward regression models identified RPWM Cre (β = −.43) and PCC NAA (β = −.27) in predicting Agreeableness scores (R**^2^**  = .35), supported well by AICc models. The interaction of these variables was not significant (β = −1.58).

Finally, Conscientiousness scores were predicted by LFWM Cho (β = −.38) and PCC Cre (β = .35) under both stepwise and backward regression methods (R**^2^** = .25). The interaction of these variables was not significant (β = −.57). The best AICc models use both Cho and Cre from these locations to predict Conscientiousness. All bivariate correlations between spectroscopic concentrations for NAA (Table 3), Creatine (Table 4) Choline (Table 5) and personality variables are shown. Bivariate correlations between all metabolites, across all six regions of interest, are shown in Table 6.

**Table 3:** Bivariate Correlations between Independent and Dependent Variables.

	*N*	*E*	*O*	*A*	*C*	*RFWM*	*ACC*	*LFWM*	*RPWM*	*PCC*	*LPWM*
Neuroticism	**-**										
Extraversion	−.39**	-									
Openness	−.06	.06	-								
Agreeableness	−.13	.20	.04	-							
Conscientiousness	−.29*	.18	−.22	.24	-						
RFWM	.16	−.07	−.19	−.23	−.16	-					
ACC	.02	.16	.02	.03	.08	−.25	**-**				
LFWM	.12	.10	−.02	−.02	−.13	.38**	.26*	**-**			
RPWM	.27*	−.15	−.03	−.19	−.31*	.79**	−.25	.24	**-**		
PCC	−.19	.15	.03	−.04	.15	−.23	.50**	−.02	−.22	**-**	
LPWM	.24	.05	.00	−.06	−.24	.31*	.36**	.72**	.37**	.06	**-**

Correlation coefficients and significance (* = p<.05; ** = p<.01) for the NEO-FFI and NAA Concentrations.

RFWM–right frontal white matter; dACC–dorsal anterior cingulate cortex; LFWM–left frontal white matter; RPWM–right parietal white matter; PCC–posterior cingulate cortex; LPWM–left parietal white matter.

**Table 4:** Bivariate Correlations between Creatine concentrations and Personality Variables.

	*N*	*E*	*O*	*A*	*C*	*RFWM*	*ACC*	*LFWM*	*RPWM*	*PCC*	*LPWM*
Neuroticism	-										
Extraversion	−.39**	-									
Openness	−.06	.06	-								
Agreeableness	−.13	.20	.04	-							
Conscientiousness	−.29*	.18	−.22	.24	-						
RFWM	.14	−.08	−.08	−.28*	−.11	-					
ACC	−.10	.09	.12	−.03	.12	−.13	-				
LFWM	.03	.02	−.00	−.10	−.02	.03	.30*	-			
RPWM	.12	−.29*	−.05	−.35**	−.19	.74**	−.14	.04	-		
PCC	−.23*	.11	−.01	−.04	.35**	−.15	.29*	.14	−.18	-	
LPWM	.00	−.12	−.00	−.07	.00	.24	.31*	.57*	.36*	.23	-

Correlation coefficients and significance (* = p<.05; ** = p<.01), for the NEO-FFI and Cre Concentrations.

RFWM–right frontal white matter; dACC–dorsal anterior cingulate cortex; LFWM–left frontal white matter; RPWM–right parietal white matter; PCC–posterior cingulate cortex; LPWM–left parietal white matter.

**Table 5:** Bivariate Correlations between Choline concentrations and Personality Variables.

	*N*	*E*	*O*	*A*	*C*	*RFWM*	*ACC*	*LFWM*	*RPWM*	*PCC*	*LPWM*
Neuroticism	-										
Extraversion	−.39**	-									
Openness	−.06	.06	-								
Agreeableness	−.13	.20	.04	-							
Conscientiousness	−.29*	.18	−.22	.24	-						
RFWM	.03	−.23	−.07	−.26*	−.31*	-					
ACC	−.03	−.09	.14	−.10	.00	.12	-				
LFWM	.08	−.18	.08	−.30*	−.21*	.68**	.42**	-			
RPWM	.12	−.20	−.01	−.21*	−.18	.80**	.15	.60**	-		
PCC	−.25	.12	.03	−.05	.16	.27*	.39**	.12	.29*	-	
LPWM	.10	−.13	.01	−.13	−.13	.54**	.42**	.68**	.71**	.45**	-

Correlation coefficients and significance (* = p<.05; ** = p<.01), for the NEO-FFI and Cho Concentrations.

RFWM–right frontal white matter; dACC–dorsal anterior cingulate cortex; LFWM–left frontal white matter; RPWM–right parietal white matter; PCC–posterior cingulate cortex; LPWM–left parietal white matter.

**Table 6:** Bivariate Correlations between N-acetylaspartate, Creatine, and Choline across all voxel locations.

	*NAA1*	*NAA2*	*NAA3*	*NAA4*	*NAA5*	*NAA6*	*Cfe1*	*Cre2*	*Cre3*	*Cre4*	*Cre5*	*Cre6*	*Cho1*	*Cho2*	*Cho3*	*Cho4*	*Cho5*	*Cho6*
NAA1	1																	
																		
NAA2	−.248	1																
	.056																	
NAA3	**.** ***384***	**.** ***258***	1															
	**.** ***002***	**.** ***046***																
NAA4	**.** ***794***	−.247	.237	1														
	**.** ***000***	.057	.069															
NAA5	−.229	**.** ***501***	−.021	−.221	1													
	.079	**.** ***000***	.871	.089														
NAA6	**.** ***310***	**.** ***357***	**.** ***722***	**.** ***371***	.060	1												
	**.** ***016***	**.** ***005***	**.** ***000***	**.** ***004***	.651													
Cre1	**.** ***515***	***−.363***	−.071	**.** ***442***	***−.471***	−.073	1											
	**.** ***000***	**.** ***004***	.587	**.** ***000***	**.** ***000***	.581												
Cre2	−.192	**.** ***640***	.229	***−.299***	.081	.232	−.126	1										
	.141	**.** ***000***	.079	**.** ***020***	.537	.075	.338											
Cre3	.000	−.004	**.** ***407***	−.056	−.205	**.** ***381***	.031	**.** ***300***	1									
	1.000	.974	**.** ***001***	.671	.117	**.** ***003***	.815	**.** ***020***										
Cre4	**.** ***554***	***−.386***	−.057	**.** ***646***	***−.480***	.044	**.** ***736***	−.135	.036	1								
	**.** ***000***	**.** ***002***	.666	**.** ***000***	**.** ***000***	.737	**.** ***000***	.303	.785									
Cre5	−.194	−.037	−.141	***−.432***	**.** ***420***	***−.271***	−.149	**.** ***292***	.140	−.175	1							
	.138	.781	.284	**.** ***001***	**.** ***001***	**.** ***036***	.257	**.** ***024***	.286	.181								
Cre6	.069	−.097	.247	.015	−.217	**.** ***387***	.235	**.** ***305***	**.** ***568***	**.** ***358***	.232	1						
	.602	.461	.057	.910	.096	**.** ***002***	.071	**.** ***018***	**.** ***000***	**.** ***005***	.075							
Cho1	**.** ***552***	***−.295***	−.063	**.** ***507***	−.147	−.053	**.** ***531***	−.174	−.168	**.** ***564***	.058	.073	1					
	**.** ***000***	**.** ***022***	.630	**.** ***000***	.262	.688	**.** ***000***	.185	.199	**.** ***000***	.660	.579						
Cho2	−.143	**.** ***672***	.035	−.127	.253	.210	−.178	**.** ***693***	.006	−.012	.123	.131	.123	1				
	.276	**.** ***000***	.789	.333	.051	.107	.173	**.** ***000***	.962	.927	.351	.317	.349					
Cho3	**.** ***322***	−.067	.072	**.** ***283***	−.151	.215	.204	.145	.229	**.** ***367***	.104	**.** ***315***	**.** ***682***	**.** ***422***	1			
	**.** ***012***	.610	.584	**.** ***028***	.248	.099	.117	.270	.079	**.** ***004***	.430	**.** ***014***	**.** ***000***	**.** ***001***				
Cho4	**.** ***499***	***−.299***	−.078	**.** ***617***	−.223	.056	**.** ***458***	−.148	−.055	**.** ***692***	−.037	.214	**.** ***799***	.147	**.** ***595***	1		
	**.** ***000***	**.** ***020***	.555	**.** ***000***	.087	.672	**.** ***000***	.258	.675	**.** ***000***	.780	.101	**.** ***000***	.264	**.** ***000***			
Cho5	−.122	.196	−.152	−.126	**.** ***634***	−.112	−.173	.073	−.106	−.084	**.** ***573***	.022	**.** ***265***	**.** ***390***	.195	**.** ***294***	1	
	.351	.134	.246	.337	**.** ***000***	.394	.186	.578	.420	.524	**.** ***000***	.869	**.** ***041***	**.** ***002***	.135	**.** ***023***		
Cho6	.102	−.064	−.038	.139	−.095	.157	.165	.165	.118	**.** ***385***	.213	**.** ***497***	**.** ***544***	**.** ***420***	**.** ***680***	**.** ***709***	**.** ***445***	1
	.440	.626	.774	.290	.472	.232	.207	.207	.371	**.** ***002***	.102	**.** ***000***	**.** ***000***	**.** ***001***	**.** ***000***	**.** ***000***	**.** ***000***	

Bold/italic font: Correlation is significant at p<0.05 level (2-tailed).

NAA  =  N-acetylaspartate; Cre  =  Creatine; Cho  =  Choline; 1 =  right frontal white matter; 2 =  anterior cingulate cortex; 3 =  left frontal white matter; 4 =  right parietal white matter; 5 =  posterior cingulate cortex; 6 =  left parietal white matter

Due to the high correlation between Neuroticism and Extraversion (−.39) in our sample, and the fact that both were related to PCC Cho in our AICc models, we tested whether these effects remained significant when controlling for personality variables in backwards regression in a *post hoc* manner. When controlling for Extraversion, the relationship between Neuroticism and PCC Cho remained significant (p = .02); however, when controlling for Neuroticism, the relationship between Extraversion and PCC Cho was no longer significant (p = .17). Thus, the PCC Cho relationship to personality appears to be specific to Neuroticism as opposed to Extraversion in our sample.

## Discussion

Our study found that individual variation in neurochemical concentrations detected by ^1^H-MRS was related to specific aspects of personality functioning in a cohort of normal human subjects. For the purpose of this discussion, we will broadly refer to each metabolite in terms of its major functional role: NAA as a neuronal marker, Cre as a marker of tissue energetics, and Cho as a marker of cellularity. For example, higher NAA has commonly been associated with neuronal health, viability, and high cognitive ability in cohorts of healthy subjects [14], [15], [24]; Cre, an energy store for the brain, has also been associated with increased mental performance [25], possibly via alteration of vascular reactivity [26] or ATP synthesis [27] during cortical activation; Cho elevations have been linked with membrane turnover associated with cellular remodeling in affective disorders [28].

Many of the relationships between brain biochemistry and personality (except for Openness) were found within brain regions overlapping the so-called “Default Mode Network.” The Default Mode Network (DMN) is engaged during resting states (e.g. when an individual is left to his or her own thoughts), and is activated during times of self reflection, which include autobiographical memory retrieval, envisioning the future, and perspective taking [29], [30]. The DMN is comprised of brain regions including the medial prefrontal cortex (MPFC), PCC/retrosplenial cortex, the precuneus and the inferior parietal lobule (IPL)[29]. A recent partial correlation network analysis demonstrated that the precuneus and the PCC possess a strong level of interaction with the other nodes of the DMN, indicating a central role for these structures in the functioning of the DMN [31]. The PCC specifically, acts as a relay station integrating the medial temporal lobe subsystem and the medial prefrontal subsystem [29]. Interestingly, the PCC is involved in autobiographical memory retrieval [32], and becomes active when individuals make self-relevant, affective decisions [33].

Our findings involved differing concentrations of Cho, Cre, and NAA in regions both within (i.e., PCC cortex) and white matter underlying (i.e., precuneus) the DMN. Both positive and negative relationships were found between levels of Cho and Neuroticism, Extraversion, and Conscientiousness. We have previously hypothesized a link between Cho concentrations, myelin turnover, and efficiency of white matter functioning [14]; thus lower Cho in the LAWM and dACC gyrus could reflect more efficient white matter functioning and/or lower myelin turnover rates in subjects with high Conscientiousness and Extraversion respectively. The inverse correlation in the LAWM Cho and Conscientiousness replicates a significant relationship between Conscientiousness and the volume of a region of lateral PFC extending across most of the left middle frontal gyrus [34]. Similarly, the inverse relationship relates to our previous finding in which we found inverse relationships between Cho obtained within the LAWM and positive affect in young cohort [18]. In contrast, Neurotic subjects differed significantly in the direction with which PCC Cho predicted their respective personality domain, reflecting lower PCC membrane turnover. Our findings correspond well with the predominantly medial and inverse structural findings reported previously [35], with our Neuroticism results revealing a biochemical profile predominantly reflective of membrane turnover and remodeling associated with the Cho resonance.

The specific relationships we found between Cre in either the PCC or the precuneus and each of the personality traits indicate a complex relationship between personality, tissue energetics, and the DMN. For example, low concentrations of Cre in the right precuneus predicted Agreeableness and Extraversion. Based on the fact that Cre, an energy reserve, stores phosphate which is utilized when the cell has used up its ATP—we hypothesize that higher concentrations of Cre are necessary when a neuron is engaged in longer high energy activity within the cell. Thus, low concentrations of Cre in the right precuneus could be indicative of 1) shorter activation periods and/or 2) less energetically intense periods of activation in individuals high in Agreeableness and/or Extraversion. Previous investigation into the precuneus' functional role demonstrated that it is involved in self-centered mental imagery strategies during rest, and the network of neural correlates of self-consciousness [36], [37]. Interestingly, our results would suggest that the precuneus might require less metabolic reserve in individuals who rank high in Agreeableness and Extraversion. In contrast, higher Cre, found in the PCC of Conscientious subjects and the dACC of Extraverted subjects, would suggest longer and/or more intense activation of these structures in service of personality demands.

Finally, we found that NAA was related to several personality domains, particularly Neuroticism, Openness, and Agreeableness. Most interesting was the relationship of NAA with Openness, with a positive relationship being found between right parietal white matter NAA and an inverse pattern being found between left frontal NAA. One of the main facets of Openness is Intellect, and this personality domain is highly correlated with measured intelligence [38]. Interestingly, we previously found that left parietal white matter NAA was predictive of intelligence [14], while left frontal NAA was inversely related to intelligence [12]: very similar to the pattern demonstrated with Openness. Similarly, higher NAA was also seen in the right parietal white matter in Neuroticism, suggesting higher levels of neuronal integrity in this brain region. Neuroticism has also been associated with intelligence, although negatively, a result considered to be mediated significantly by test anxiety [39]. Interestingly, high intelligence is often seen to buffer against the negative performance characteristics of Neuroticism [40], a characteristic reflected in our high intelligence sample (Mean  = 118). Too, our sample had lower than average Neuroticism (Mean  = 16.43), which is at the 15^th^%ile for a college sample. Future research exploring the systematic relationship between neuronal integrity (e.g., NAA), Neuroticism, and intelligence would appear to be fruitful based on the current results in a relatively high IQ cohort.

There are several limitations to this study. First, we have employed a technique which does not assess brain biochemistry across the entirety of the brain, but within a limited area, within the supraventricular region, sampling predominantly white matter underlying the frontal and parietal lobes, and central grey matter comprising the cingulate gyrus. This prevents us from making strong comparisons to previous structural personality studies conducted outside of areas beyond our spectroscopic region of interest, although we have attempted to extend our findings to projection zones to which our white matter voxels are logically connected. This may be erroneous, as we have not conducted simultaneous diffusion tensor imaging to determine specific white matter tracts that might pass through our voxels of interest. Second, our sample is both young and of above average intelligence, which is common in samples obtained from college undergraduate communities. The generalizability of these findings to other populations, particularly older cohorts is unknown given the rather constrained age of our subjects. Third, the spectroscopic variables are intercorrelated, raising issues of multi-collinearity (Table 6). There is a discussion in the literature but no consensus regarding how best to deal with collinear variables in modeling research [41], [42]. As this is exploratory research in personality neuroscience, applying spectroscopic methods to this nascent discipline for the first time, we chose to defer this issue until the implications of different methods are clearer. Finally, our biochemical assay of NAA, Cho, and Cre is limited by the constraints of both the echo time we chose to use (rather long) and the field strength of our magnet (1.5 Tesla). Other metabolites, particularly glutamate, glutamine, and inositol, which can be obtained reliably at shorter echo times and increased field strengths, would be of keen interest to personality neuroscience. Glutamate and glutamine, for example, are measures of neurotransmission between neurons, and recycling of energetic byproducts through astrocytes ; inositol is considered to be a glial cell marker, much as NAA is considered to be a marker of neuronal mass and viability [43].

Recent studies have begun to discern the functioning of the DMN, but will require extensive investigation to provide the necessary evidence to determine the role of the DMN functioning in personality. Our results suggest that variation in neuronal density, cellularity, and energetics in the areas of the DMN is related to most of the major personality domains as measured by the NEO. Our results are a large step forward for personality neuroscience as we begin to tease apart the structural, functional, and biochemical systems involved in normal personality functioning.
